# Evidence for an increased distribution range of *Dermacentor reticulatus* in south-west Poland

**DOI:** 10.1007/s10493-012-9612-3

**Published:** 2012-10-13

**Authors:** Dorota Kiewra, Aleksandra Czulowska

**Affiliations:** Institute of Genetics and Microbiology, University of Wroclaw, Wrocław, Poland

**Keywords:** *Dermacentor reticulatus*, Poland, Geographical distribution, Change the range

## Abstract

The expansion of *Dermacentor reticulatus* to new geographical areas has been observed in several countries in Europe, including Poland and it’s neighbors. In 2011 and 2012, a total of 148 host-seeking *D. reticulatus* ticks were collected after flagging the vegetation in Lower Silesia, south-western Poland. Tick monitoring was conducted in mixed and deciduous forest, on meadows, in river valleys and ecotones between forested and grassy areas. The ornate dog ticks were found in 10 out of 33 sites located in five districts: Legnica, Wroclaw, Środa Śląska, Lubin and Boleslawiec County. All sites where *D. reticulatus* ticks were found are located to the south-west of the Odra River. The greatest distance between these disconnected localities was approximately 90 km. It seems that at present the southern boundary of the range is Wroclaw district. This study indicates that *D. reticulatus* can be ranked as a typical element of the fauna in Lower Silesia in southwestern Poland.

## Introduction

There are 36 known *Dermacentor* tick species worldwide (Barker and Murrell 2008), and two of them have been found in Poland: *Dermacentor reticulatus* (the ornate dog tick or the ornate cow tick) and *D. marginatus* (the ornate sheep tick). According to Siuda (1993) *D. marginatus* was collected in Poland only from wild boar (*Sus scrofta*) (only a few specimens in the Kłodzko Valley, Lower Silesia, south-western Poland) and it can be consider as a transferred species (Nowak 2010a), whereas *D. reticulatus* was commonly found in north-eastern Poland. Taking into account habitats of mature tick stages it can be classified as non-nidicolous, whereas nymphs and larvae attack small mammals in burrows and can be classified as nidicolous. Adults feed on larger mammals like deer and dogs but only occasionally bite humans (Dautel et al. 2006). Consequently, immature stages were only found on hosts, whereas adults can be collected both from the hosts and the vegetation. In Poland, *D. reticulatus* ticks inhabit mostly river valleys, lake shores, meadows and clearings, preferring natural boggy mixed and deciduous forests connected to a watercourse, where there is a relatively high ground water table (Siuda 1993; Karbowiak 2009).

The distribution of the ornate dog tick is known in the western Palearctic region, in a temperate climate zone from England and France in the west, to the basin of Yenisei River in Siberia in the east (Siuda 1993). It has not been found north of 53–54°N latitude nor in the Mediterranean climate zone (Dautel et al. 2006). However, a case of babesiosis caused by *Babesa canis canis* in a dog in Norway suggest possibility of expansion *D. reticulatus* even further northwards (Øines et al. 2010). Within the geographic range, the ornate dog tick, is distributed in a highly focal pattern (Gray et al. 2009). However, the range of the ornate dog tick occurrence is divided into two separate localities. In western Europe it includes areas in England, France, Switzerland, Germany, Austria, whereas in eastern Europe it extends from north-eastern Poland across Lithuania, Belarus, Ukraine to the European part of Russia. Therefore, the western part of Poland was considered to be a region free from *D. reticulatus*. Karbowiak (2009) indicates the triangular sharp area, where *D. reticulatus* has been absent. The north border of this triangle ranges along the Baltic seaside, whereas the arms extended from central Germany, from the 12–13th meridian longitude East to the 19th meridian in Poland, and coincides with the southern border of Hungary. However, in recent years, the range of this species has been extended substantially and new localities of *D. reticulatus* have been found.

The present study for the first time shows that *D. reticulatus* can be ranked as a typical element of the fauna in Lower Silesia in south-western Poland.

## Materials and methods

**Fig. 1:**
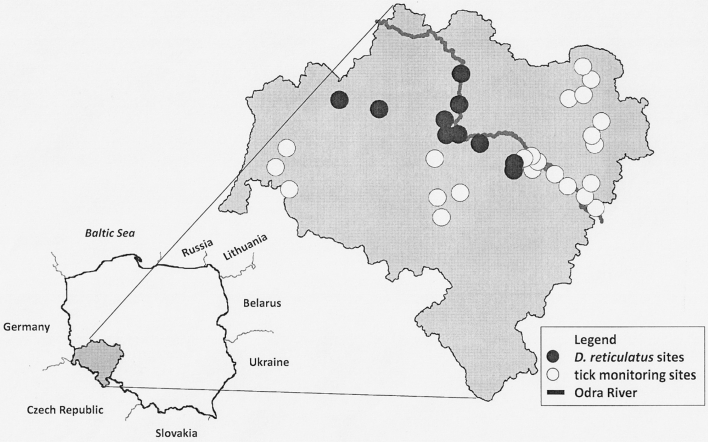
Tick monitoring habitats and collection sites of *Dermacentor reticulatus* in Lower Silesia, Poland

Host-seeking ticks were collected using a flagging method in Lower Silesia, in south-western Poland. The study was conducted in 33 localities from Godnowa (N51°33.460 E017°18.986) in the North to Świebodzice (N50°54.412 E016°23.948) in the South (Fig. 1). Different type of habitats were selected: in 2011 (April–June and September), sampling of ticks was carried out in habitats preferred by *Ixodes ricinus* ticks (mixed and deciduous forest), while in 2012 (March–April), tick monitoring was conducted also on meadows, in the river valleys, and ecotones between forested and grassy areas. Ticks were collected in daytime between 9 a.m. and 5 p.m. Each single site was flagged for at least 30 min. Collected ticks were stored in 70 % ethanol. Adult ticks were separated by sex, and identified to the species level under stereomicroscope by using the key in a monographic work of Siuda (1993).

## Results

A total of 148 *D. reticulatus* ticks were collected from vegetation in 10 out of 33 sites in Lower Silesia, south-western Poland (Fig. 1; Table 1). In 2011, ten host-seeking *D. reticulatus* ticks (5 female and 5 male) were collected using a flagging method, among *I. ricinus* from three sites located in the Legnica County (Mierzowice, Motyczyn, Rogów Legnicki). In 2012, 138 questing *D. reticulatus* ticks (84 female and 54 male) were found. In 2012, the occurrence of *D. reticulatus* ticks was confirmed in all three sites selected in 2011, and additionally in seven new sites located in five districts: the Legnica County (Rokitki), the Lubin County (Naroczyce, Dziewiń), the Wroclaw County (Wroclaw, Samotwór), the Bolesławiec County (Trzebień), the Środa Śląska County (Szczepanów). All sites where *D. reticulatus* ticks were found are located to the south-west of the Odra River. The distance between the most distant localities, where *D. reticulatus* ticks were found, is approximately 90 km.

**Table 1:** The number of *Dermacentor reticulatus* ticks collected in 10 sites in Lower Silesia in years 2011–2012

Site no.	Years	Locality	Geographical coordinates	Number of *D. reticulatus*		Density^a^
				Females	Males	
1	2011	Motyczyn	N51°14.367 E 016°21.732	1	0	1
2		Mierzowice	N 51°17.787 E 016°20.802	3	5	4
3		Rogów Legnicki	N 51°14.726 E 016°26.887	1	0	0.5
1	2012	Motyczyn	N 51°14.367 E 016°21.732	8	4	4
2		Mierzowice	N 51°17.787 E 016°20.802	14	13	5.4
3		Rogów Legnicki	N 51°14.726 E 016°26.887	6	1	2.3
4		Naroczyce	N 51°31.246 E 016°27.247	41	33	12.3
5		Dziewin	N 51°21.725 E 016°27.948	2	0	0.7
6		Wrocław (Jarnoltow)	N 51°07.555 E 016°50.409	3	1	4
7		Szczepanow	N 51°12.320 E 016°37.355	3	1	4
8		Trzebień	N 51°22.033 E 015°36.015	3	1	2
9		Samotwór	N 51°06.562 E 016°49.957	1	0	0.5
10		Rokitki	N 51°19.603 E 015°53.603	3	0	1.5

^a^ The average number of *D. reticulatus* ticks collected by one person during 30 min

## Discussion

The expansion of *D. reticulatus* into new areas has been observed in several European countries, including Poland and it’s neighbouring states. Climate change, increased deer abundance, and antropogenic factors together with more fallow land may be responsible for the change in the distribution (Gray et al. 2009). Colonization of new sites is known from Germany (Dautel et al. 2006; Gray et al. 2009), the Netherlands (Nijhof et al. 2007), Slovakia (Bullová et al. 2009), the Czech Republic (Široký et al. 2011). In Germany, *D. reticulatus* has expanded its range particularly in the eastern and southwestern parts of country (Gray et al. 2009). Dogs and deer infested with *D. reticulatus* as well as specimens in the field, were found in Brandenburg. In Saxony deer were found to be harbouring *D. reticulatus* (Dautel et al. 2006). Thus, the ornate dog ticks were found in the German land bordering western Poland. On the other hand, the closest position of *D. reticulatus* ticks to the south-west border of Poland is confirmed only in south-east part of the Czech Republic in the South Moravian Region, where north-west distribution limits of this species were defined (Široký et al. 2011). In Poland, the occurrence of *D. reticulatus* ticks is known, however only a north-east part of the country is considered as a typical location. Earlier and present works confirm locations mainly in the Warmian-Masurian, the Podlaskie, and the Lublin Voivodship, along the border with Lithuania, Belarus, and Ukraine (Szymański 1977; Siuda 1993; Bogdaszewska 2005; Biaduń et al. 2007; Bartosik et al. 2011). However, the expansion towards west is observed for several years. *D. reticulatus* ticks were noted in the Pomeranian, the Kuyavian-Pomeranian, and West-Pomeranian Voivodship to the West of the Vistula River (Fryderyk 1998, Kadulski and Izdebska 2009), in the Masovian Voivodeship (Karbowiak 2009). In the present study, new sites of *D. reticulatus* ticks in south-western Poland were found. The first evidence of the ornate dog tick occurrence in Lower Silesia was noted in 2009, when a few species in the Boleslawiec County were collected (Karbowiak and Kiewra 2010). The Boleslawiec locality as *D. reticulatus* tick colonized area was confirmed in the present study. In addition, the *D. reticulatus* was found on 9 additional sites, including three sites where this species was collected both in 2011 and 2012. This results indicate the constant presence of this species in Lower Silesia. The occurrence of *D. reticulatus* in Western Poland was also recently discovered in 2010 in the Lubuskie Province as a “Lubuskie Focus”, about 55 km from the Polish-German border (Nowak 2010b). Taking into account the present study (the distance between sites was about 90 km) and the 70 km distance between currently data from Naroczyce, the most northerly located position in 2012, and “Lubuskie Focus” it is necessary to consider the change of the *D. reticulatus* range. What’s more it may be the extension of the West European population. It seems that at present the south boundary of the range is Wrocław district.

The knowledge of tick distribution is particularly important from a medical and veterinary point of view because of a variety of pathogens transmitted, including viruses, bacteria and protozoa. *D. reticulatus* ticks are known to transmit *Babesia* spp., *Rickettsia* spp., *Francisella tularensis*, *Coxiella burnetii*. Endemic regions for canine babesiosis caused by *B. canis* are the same as endemic regions for *D. reticulatus* (Zygner et al. 2009). In Slovakia, according to Bullová et al. (2009) the occurrence of babesiosis preceded the founding of *D. reticulatus* in the East Slovak Lowland. Also the occurrence of canine babesiosis in new areas of Germany, Hungary, Switzerland and the Netherlands provided the changing distribution of the ornate dog ticks (Gray et al. 2009). Simultaneously with the appearance of *D. reticulatus* in Lower Silesia, there is danger of spreading not only canine babesiosis, but also other tick-borne diseases like rickettsiosis. In Germany, Dautel et al. (2006) detected almost one quarter of the *D. reticulatus* positive for *Rickettsia* sp. and in Saxony, Silaghi et al. (2011) over 65 %. A high proportion of infected ticks (over 40 %) was found also in north-eastern Poland (Stańczak 2006). In Poland, the first case of ricketsiosis TIBOLA/DEBONEL was described in 2011 (Chmielewski et al. 2011). The expansion of ticks in new areas may also cause the appearance of tick-borne encephalitis virus. According Wójcik-Fatla et al. (2011) the infection rate with TBEV of *D. reticulatus* in eastern Poland was much higher than the infection rate of *I. ricinus*.

The expansion of ticks to new area can increase a risk of tick-borne diseases. Thus, the environmental monitoring of changes in the range of *D. reticulatus* is crucial for assessment the epidemiological risk. The confirmed occurrence of the ornate dog ticks in south-west Poland provided evidence that this species has extended its range. It indicates a need of future systematic sampling to assess a speed of spread.
